# Bioprospecting of culturablebactéria with potential polyhydroxyalkanoates (PHA) producer, isolated from contaminated sites of Manaus-AM/Brazil

**DOI:** 10.1186/1753-6561-8-S4-P251

**Published:** 2014-10-01

**Authors:** Jorge Luis Manrique, Antônia QL Souza, Jair Max F Maia

**Affiliations:** 1 Universidade do Estado do Amazonas, São José, AM, Brazil

## 

Polyethylene and polypropylene, known as conventional plastic, from non-renewable sources, like oil, are successfully replacing by glasses and papers in packaging industry due to their low cost, variety and durability [1]. However, these plastics for being slow degradability and hinder the exchange of gas and the decomposition of other compounds cause health problems when they are not disposed of properly [2].

Nowadays, there are another renewable plastic sources, known as polyhydroxyalkanoates (PHA), manufactured by plants, fungi and/or bacteria source. From the ecological view this plastics are important because their degradation are faster and requires less energy in their manufacture processes, reducing significantly the CO_2 _emissions [3].

The subject of this work was a bioprospect culturable bacteria potentially producers of PHAs, from water and plant species contaminated from anthropogenic actions.

Bacteria were isolated from two plants (Superfamily Legominosae) and water collected from two points of Forty affluent of the city of Manaus - Amazonas. For the isolation of the water samples, 50 l were grown in two culture media (Nutrient Agar Agar and Lb) containing 50 mg / mL of fluconazole at 26 oC for 7 days. The plant samples (leaf, stem and root) were subjected to aseptic procedure described by Souza et al, 2004 [4]. Isolated bactéria were purified by the method of cross-striations and then evaluated by analyzing the morphology of the Gram stain.

For detection of potentially producing PHA bacteria it was used the test of Sudan Black plating cell culture with 24 wells containing YM medium and incubated at 26°C for 72 hours according to the protocol described by the National Center for Biotechnology Education (2012 [5]).

Of the four samples collected at two points of Igarapé do Quarenta, 80 bacteria were isolated, of which 64 were randomly selected and evaluated for potential production of PHA. From all bacteria evaluated, 13 were positive (30%), six (9.37%) of the plant #2, three (4.71%) of the plant #1 and two (3.12%) of each point of colleted water. From the 13 positive results, 12 were characterized as Gram negative and Gram negative as streptococci isolated from the root of the plant 1. This research showed a predominantly Gram-negative microbiota, whose endophytic bacteria were the main source, compared with water of potentially producing of PHA bacteria and therefore the bacteria present in polluted waters and adjacent plants of Igarapé do Quarenta in the city of Manaus may be considered potentially producing PHA.
